# Activity-Based Anorexia Dynamically Dysregulates the Glutamatergic Synapse in the Nucleus Accumbens of Female Adolescent Rats

**DOI:** 10.3390/nu12123661

**Published:** 2020-11-28

**Authors:** Francesca Mottarlini, Giorgia Bottan, Benedetta Tarenzi, Alessandra Colciago, Fabio Fumagalli, Lucia Caffino

**Affiliations:** Department of Pharmacological and Biomolecular Sciences, Università degli Studi di Milano, Via Balzaretti 9, 20133 Milano, Italy; francesca.mottarlini@unimi.it (F.M.); giorgia.bottan@unimi.it (G.B.); benedetta.tarenzi@unimi.it (B.T.); alessandra.colciago@unimi.it (A.C.); fabio.fumagalli@unimi.it (F.F.)

**Keywords:** nucleus accumbens, reward circuit, glutamatergic receptors, activity-based anorexia, adolescence, female rats

## Abstract

Intense physical activity and dieting are core symptoms of anorexia nervosa (AN). Their combination evolves into compulsivity, leading the patient into an out-of-control spiral. AN patients exhibit an altered activation of nucleus accumbens (NAc), revealing a dysfunctional mesocorticolimbic reward circuitry in AN. Since evidence exists that a dysregulation of the glutamate system in the NAc influences reward and taking advantage of the activity-based anorexia (ABA) rat model, which closely mimics the hallmarks of AN, we investigated the involvement of the glutamatergic signaling in the NAc in this experimental model. We here demonstrate that food restriction causes hyperactive and compulsive behavior in rodents, inducing an escalation of physical activity, which results in dramatic weight loss. Analysis of the glutamate system revealed that, in the acute phase of the pathology, ABA rats increased the membrane expression of GluA1 AMPA (α-amino-3-hydroxy-5-methyl-4-isoxazolepropionic acid) receptor subunits together with its scaffolding protein SAP97. Recovery of body weight reduced GluN2A/2B balance together with the expression of their specific scaffolding proteins, thus suggesting persistent maladaptive neurotransmission. Taken together, AMPA and NMDA (N-methyl-D-aspartate) receptor subunit reorganization may play a role in the motivational mechanisms underlying AN.

## 1. Introduction

Anorexia nervosa (AN) is a severe life-threatening psychiatric disorder with the highest rate of mortality among psychiatric disorders [1,2,3]. AN develops around the onset of puberty [4,5,6] and it is characterized by an extreme regimen of restricted diet and intense physical exercise to lose weight [7]. Anorexic patients refuse to maintain a normal body-weight status and persist in losing mass, even though they are severely emaciated. According to the literature, 40–80% of AN patients show excessive levels of physical activity, described as hyperactivity, characterized by a compulsive need to exercise [8,9]. Malnutrition and hyperactivity drive AN patients into an out-of-control spiral in which the positive feeling of complete control over food intake and body shape becomes the only reward, continuously reinforcing anorexigenic behaviors, i.e., hyperactivity and dieting.

Recently, it has been proposed that these two features of AN are initially goal-directed, based on instrumental learning, and driven by the desired outcome of weight loss [10,11]. Over time malnutrition via disruption of hormonal regulation, normal puberty trajectory and brain maturation drive a gradual shift from goal-directed to habit formation and compulsive behaviors despite increasingly negative consequences [12,13]. This behavioral shift might involve a maladaptive reorganization of the mesolimbic reward system, similar to the events that drive the transition from hedonic to compulsive drug use [14]. In addition, an unbalance between reward and inhibition systems has been proposed as neurobiological underpinning of AN [15,16] pointing to the nucleus accumbens (NAc, the ventral part of the striatum) as a brain region crucially involved in integrating reward and motivational aspects relevant to feeding behavior [17].

In line with this hypothesis, fMRI data reveal that adolescent patients affected by AN show increased activation of the ventral striatum activity upon underweight stimuli and, decreased activation upon normal-weight stimuli versus healthy control [18]. Of note, similar findings were confirmed in the activity-based animal model of AN (ABA), in which the chemogenetic activation of the mesolimbic reward pathway, projecting from the ventral tegmental area (VTA) to the NAc, attenuates body weight loss and delays the establishment of ABA endophenotype [19,20].

Alongside the involvement of the dopaminergic innervation in modulating the salience of food reward [21], physical activity [22] and vulnerability to ABA [23], little is known about the involvement of glutamate in reward-related brain areas in the development of pathological weight loss. The NAc integrates both dopaminergic and glutamatergic inputs, along with local GABAergic control, to refine motivated behavioral outcomes [24]. Glutamatergic plasticity in this area is a key mediator of enhanced motivation for addictive drug-seeking and incubation of drug craving [25], strengthening the hypothesis that alterations of the NAc synaptic plasticity in AN might play a crucial role in regulating starvation-related compulsive behaviors, promoting all the actions responsible for the reiteration of maladaptive responses to salient stimuli. Akin to this hypothesis, it has been demonstrated that food-restriction, as observed after long-term withdrawal from psychostimulant self-administration [26], increases surface expression of GluA1-, but not GluA2-, containing AMPA (α-amino-3-hydroxy-5-methyl-4-isoxazolepropionic acid) receptors [27], suggesting that synaptic incorporation of GluA2-lacking Ca^2+^-permeable AMPA receptors, by altering synaptic strength and transmission, may underpin the development and persistence of starvation dependence. Moreover, glutamate levels are reduced in the frontal regions of anorexic adolescent patients [28], suggesting that malnutrition may cause hypoglutamatergia, thus contributing to the establishment of AN-induced maladaptive behaviors.

Thus, the main goal of this work was to investigate the involvement of the glutamate synapse in the anorexigenic behaviors of self-starvation and hyperactivity in the NAc, as one of the fundamental brain areas of the reward system responsible for bringing motivation into action [24]. To this end, we induced anorexia nervosa in female adolescent rats by means of the well-established ABA model, originally proposed by Routtenberg and Kuznesof in 1967 [29], which was set up according to the literature [30]. Rodents were exposed to food restriction or exercise alone or to the combination of both conditions during adolescence, to dissect the specific contribution of low food intake and running wheel activity on the maintenance of ABA phenotype. Animals were sacrificed at two different time points: (1) during the acute phase of the pathology, once they achieved the anorexic phenotype (represented by the 25% of weight loss and wheel activity increasing over days), to evaluate the anorexic-induced alterations and (2) after a period of body weight recovery, without any more food deprivation or wheel activity, to identify possible molecular scars that might persist in the brain even when the bodyweight is restored. From a molecular standpoint, we investigated the expression of critical determinants of the glutamate synapse, focusing our attention particularly on the main α-amino-3-hydroxy-5-methyl-4-isoxazolepropionic acid (AMPA) and N-methyl-D-aspartate (NMDA) receptor subunits as well as on their respective scaffolding proteins, SAP97 and GRIP for AMPA receptors and SAP102 and PSD95 for NMDA glutamate receptor, which maintains glutamate receptors at the postsynaptic membrane of excitatory synapses [31].

## 2. Materials and Methods

### 2.1. Animals and Housing

Adolescent female Sprague–Dawley rats were used (Charles River, Calco, Italy). A maximum of two female siblings was taken from each litter in order to reduce “litter effects” [32] and upon their arrival were housed in groups of four rats per cage. Animals were fed with standard rat chow (ssniff Spezialdiäten GmbH, Soest, Germany) with tap water ad libitum. All efforts were made to minimize animal suffering and to maintain the lowest number of animals used: for ethical reasons, activity-based anorexia (ABA) rats were not allowed to lose more than 25% of their initial body weight.

All animal procedures were conducted at the Department of Pharmacological and Biomolecular Sciences (University of Milan, Milan, Italy), and carried out in accordance with the principles set out in the following laws, regulations, and policies governing the care and use of laboratory animals: Italian Governing Law (D.lgs 26/2014; Authorization n.19/2008-A issued 6 March 2008 by Ministry of Health); the NIH Guide for the Care and Use of Laboratory Animals (2011 edition) and EU directives and guidelines (EEC Council Directive 2010/63/UE). The experiments have been reported in compliance with the ARRIVE guidelines.

### 2.2. Experimental Design and Procedures

Adolescent female rats used in this study arrived at post-natal day (PND)28, they were grouped and housed in classical home cages with food and water ad libitum until PND 35, under standard conditions of temperature and humidity with a reversed 12 h light/dark cycle (lights on/off: 10.30 p.m./10.30 a.m.). At PND 35, animals were individually housed and randomly subdivided into four experimental groups: [1] control (CTRL) group: sedentary + food ad libitum; [2] food-restricted (FR) group: sedentary + food restriction (food limitation for 2 h/day), [3] exercise (EXE) group: voluntary running activity in a mechanical wheel + food ad libitum, and [4] activity-based anorexia (ABA) group: voluntary running activity in a mechanical wheel + food restriction (food limitation for 2 h/day). All animals started a 3 days period of habituation (PND 35-PND 38) to the new environment, in which CTRL and FR groups were maintained in classical home cages, while EXE and ABA groups were placed in specific activity cages equipped with a mechanical wheel (activity wheel BIO-ACTIVW-R cage, Bioseb, Vitrolles, France). Single housing cages were placed adjacent to each other to provide sight, acoustic and odor contact. During the habituation period, rats were exposed to daily handling to become accustomed to the interaction with the investigator. The food restriction period started at PND 38: FR and ABA rats were subjected to caloric restriction with food limited for 2 h/day from 10.30 to 12.30 a.m. and during the 2 h of food access the wheel was blocked to prevent the rats preferring to run rather than to eat. The other two groups (CTRL and EXE) continued to have free access to food for 24 h/day.

**Figure 1:**
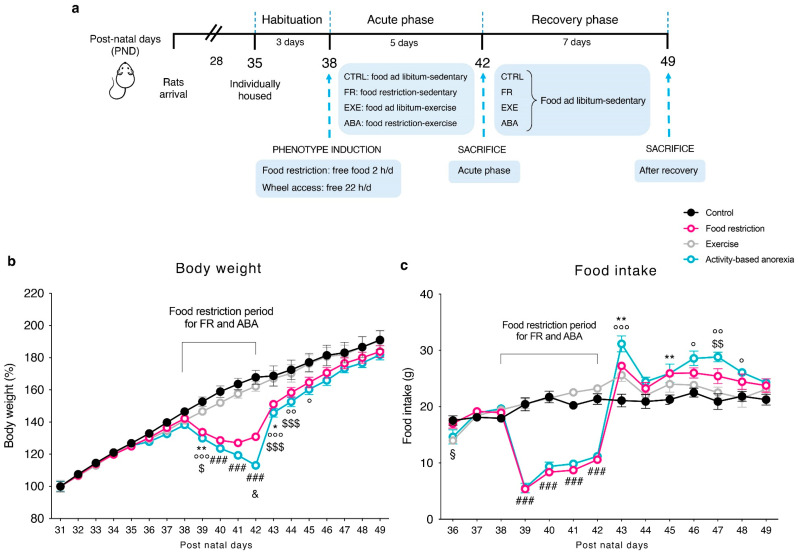
(**a**) Schematic representation of the experimental paradigm performed in adolescent female rats to induce the activity-based anorexia (ABA) phenotype; (**b**) average daily body weight; and (**c**) food intake, in control (CTRL), food-restricted (FR), exercise (EXE) and ABA rats. Results are presented as the mean ± SEM. ^###^ *p* < 0.0001 FR and ABA vs. CTRL and EXE; * *p* < 0.05, ** *p* < 0.01 CTRL vs. FR; ^°^ *p* < 0.05, ^°°^ *p* < 0.01, ^°°°^ *p* < 0.001 ABA vs. CTRL; ^$^ *p* < 0.05, ^$$^ *p* < 0.01, ^$$$^ *p* < 0.001 ABA vs. EXE; ^&^ *p* < 0.05 FR vs. ABA; ^§^ *p* < 0.05 ABA and EXE vs. CTRL and FR. (two-way analysis of variance (ANOVA) with repeated measures followed by Bonferroni’s multiple comparisons test).

At PND 42, half of the animals per group were killed by decapitation, while the remaining part was preserved sedentary with food ad libitum for 7 days until PND 49 in order to allow the bodyweight recovery. Within each experimental group, we randomly sacrificed half animals at PND 42 and the other half at PND 49. Figure 1a shows a schematic representation of the experimental paradigm herein described.

To set up the protocol 48 animals were used with 100% a rate of survival (Acute phase PND 42 *n* = 24; Recovery phase PND 49 *n* = 24; 4 experimental groups for each time point: CTRL *n* = 6, FR *n* = 6, EXE *n* = 6, ABA *n* = 6).

After decapitation, the NAc (defined as shell and core subregions) was immediately dissected from 2-mm-thick slices from Bregma + 2.76 to Bregma + 0.84 mm, corresponding to plates 11–25 of the Rat brain atlas of Paxinos and Watson [33], frozen on dry ice, and stored at −80 °C for subsequent molecular analysis.

### 2.3. Measurements

Bodyweight and food intake were assessed per each animal from the first day of habituation until the end of the experiment (3 days of habituation, 5 days of food restriction, 7 days of recovery). Rats were weighed daily between 09.30 and 10.30 a.m., before the dark shift. Food intake was measured with a specific food milligram-sensitive scale before the beginning of the 2 h of restriction and immediately after and calculated as grams of food given at the beginning of the 2 h of food access—grams weighed at the end of the 2 h.

Running wheel activity was evaluated by means of dedicated activity cages equipped with mechanical wheels (activity wheel BIO-ACTIVW-R cage, Bioseb, Vitrolles, France) connected to a counter device associated with a monitoring software (BIO-ACTIVW-SOFT v1.2.1, Bioseb, Vitrolles, France). Running wheel data were recorded at 30 min intervals for the entire duration of the experiments. Distance travelled (m), mean and maximum speed (m/min) were presented as average for each experimental day. Long-exercise sequence (number of access) was defined as the number of times per day the animal enters the wheel to run continuously for more than 2 m; whereas brief-exercise sequence (number of access) was defined as the number of times per day the animal enters the wheel to run less than 2 m.

### 2.4. Preparation of Protein Extracts and Western Blot Analyses

Proteins from NAc tissues were homogenized and the crude synaptosomal fraction was prepared as previously described [34]. Western blots on the whole homogenate and on the crude synaptosomal fraction were run as previously described [35].

The conditions of the primary antibodies were the following: GluN2A (1:1000, Cell Signaling Technology, RRID: AB_2112295); GluN2B (1:1000, Cell Signaling Technology, Danvers, MA, USA, RRID: AB_2798506); SAP97 (1:1000, AbCam RRID: Addgene_128624); GluA1 (1:1000, Cell Signaling Technology, Danvers, MA, USA, RRID: AB_2732897); GluA2 (1:1000, Cell Signaling Technology, Danvers, MA, USA, RRID: AB_10622024); SAP102 (1:1000, AbCam, Cambridge, UK, RRID: AB_2799325); PSD95 (1:3000, Cell Signaling Technology, Danvers, MA, USA, RRID: AB_2292883); GRIP (1:1000, Synaptic System GmbH, Göttingen, Germany, RRID: AB_10804287).

Results were standardized to β-actin control protein that was detected by evaluating the band density at 43 kDa after probing with a polyclonal antibody with a 1:20,000 dilution (Merck Life Sciences Srl, Milano, Italy, RRID: AB_476744). Immunocomplexes were visualized by chemiluminescence using the Chemidoc MP Imaging System (Bio-Rad Laboratories, Segrate (MI), Milan, Italy). Gels were run two times each, and the results represent the average from two different runs (the entire WB bands are presented in Supplementary Figures S1 and S2. We used a correction factor to average the different gels: correction factor gel B = average of (OD protein of interest/OD β-actin for each sample loaded in gel A)/(OD protein of interest/OD β-actin for the same sample loaded in gel B) [36].

### 2.5. Data Analysis and Statistics

Data were collected in individual animals (independent determinations) and are presented as means and standard errors. Bodyweight, food intake, distance travelled on the wheel, mean speed, maximum speed, brief- and long-exercise sequences were analyzed by two-way analysis of variance (ANOVA) with repeated measures followed by Bonferroni’s multiple comparisons test. Molecular changes were analyzed by two-way ANOVA, with physical activity (sedentary vs. exercise) and food intake (food ad libitum vs. food restriction) as independent variables. When dictated by relevant interaction terms, Tukey’s multiple comparisons test was used to characterize differences among individual groups of rats. However, when no interaction between physical activity and food intake was observed, only the main effects were reported. Detailed statistical values of data presented in each figure are reported in Supplementary Materials (Tables S1–S5).

Pearson’s product-moment coefficients were calculated to study potential correlations between behavioral outcomes induced by the ABA procedure, related to the wheel-running activity, and molecular changes observed in the NAc crude membrane fraction of ABA rodents.

Subjects were eliminated from the final dataset if their data deviated from the mean by 2 SDs. Prism 8.2.1 (GraphPad Software, Prism v8.2.1, San Diega, CA, USA) was used to analyze all the data. Significance for all tests was assumed at *p* < 0.05.

## 3. Results

Average body weight of CTRL, FR, EXE and ABA groups increased linearly, from the first experimental day (PND 31) since the beginning of the food restriction period (PND 38) (Figure 1b), with no significant differences resulting from wheel access (PND 35-PND 38). During the restriction phase, starting from PND 39 (after 24 h of food restriction), FR and ABA groups lost weight compared with CTRL and EXE groups and this difference in body weight constantly increased over days. At PND 42, ABA weight loss was significantly greater than FR, despite similar food intake (Figure 1c), highlighting the broader impact of wheel activity in accelerating bodyweight loss. During the recovery period (PND 42-PND 49, consisting of ad libitum food access for 24 h and no wheel access) both FR and ABA recovered their body weight (Figure 1b) in concomitance to an increase in food intake (Figure 1c). However, although they eat a higher amount of food, the ABA group needed more time than FR rats to completely recover their body weight at the level of CTRL, further strengthening the long-lasting influence of the ABA protocol in respect to the FR alone.

### 3.1. Food Restriction Elicited Hyperactive Running Behavior

**Figure 2:**
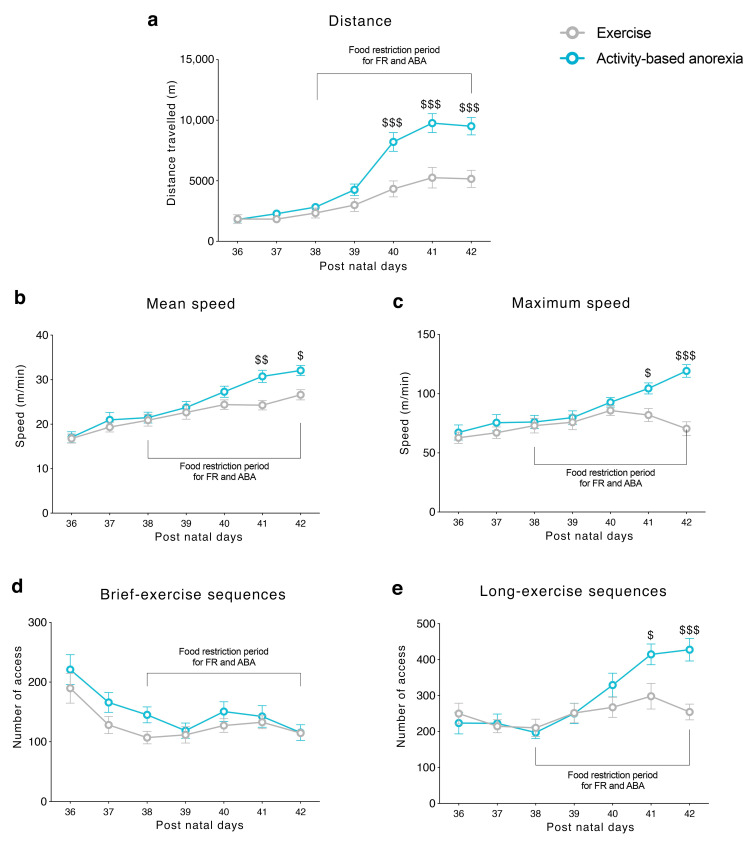
Running activity on the wheel in terms of (**a**) distance travelled in meters, (**b**) mean speed in meters/minute, (**c**) maximum speed in meters/minute, (**d**) brief- and (**e**) long-exercise sequences expressed as the total number of access lower and higher than 2 meters each respectively exhibited by EXE and ABA rats. Results are presented as the mean ± SEM. ^$^ *p* < 0.05, ^$$^ *p* < 0.01, ^$$$^ *p* < 0.001 ABA vs. EXE (two-way ANOVA with repeated measures followed by Bonferroni’s multiple comparisons test). FR = food-restricted; EXE = exercise; ABA = activity-based anorexia.

Prior to food restriction, EXE and ABA groups exhibited a stable voluntary wheel running in terms of total distance travelled, mean and maximum speed on the wheel (Figure 2a–c, respectively), reflecting their habituation to the apparatus. When the restriction schedule began, a difference in the total distance travelled between EXE and ABA rats emerged: ABA rats increased running activity reaching statistical significance by PND 40 (Figure 2a). In addition, as an index of physical exercise compulsivity, we observed an increased mean and maximum speed on the wheel in ABA rats at PND 41 and PND 42 (Figure 2b,c). Moreover, ABA rats dwelled on the wheel to perform more long-exercise sequences (more than 2 m each time they entered in the wheel) than EXE animals (Figure 2d), whereas brief-exercise sequences (less than 2 m each access on the wheel) were similar in both groups (Figure 2e).

### 3.2. Food Restriction-Evoked Hyperactivity Remodeled the Molecular Composition of the Glutamate Synapse in the NAc

In order to evaluate the impact of the anorexic phenotype on the glutamate synapse in the NAc, we examined the expression of the main glutamate AMPA and NMDA receptors and their specific scaffolding proteins in ABA animals, both at the achievement of the anorexic phenotype (acute phase, PND 42) and after a period of body weight recovery (PND 49). We investigated the expression of critical determinants of the glutamate synapse in the whole homogenate and in the crude membrane fraction to have a readout of ABA-induced effect on both translation and synaptic availability of glutamate receptors at the synaptic site.

**Figure 3:**
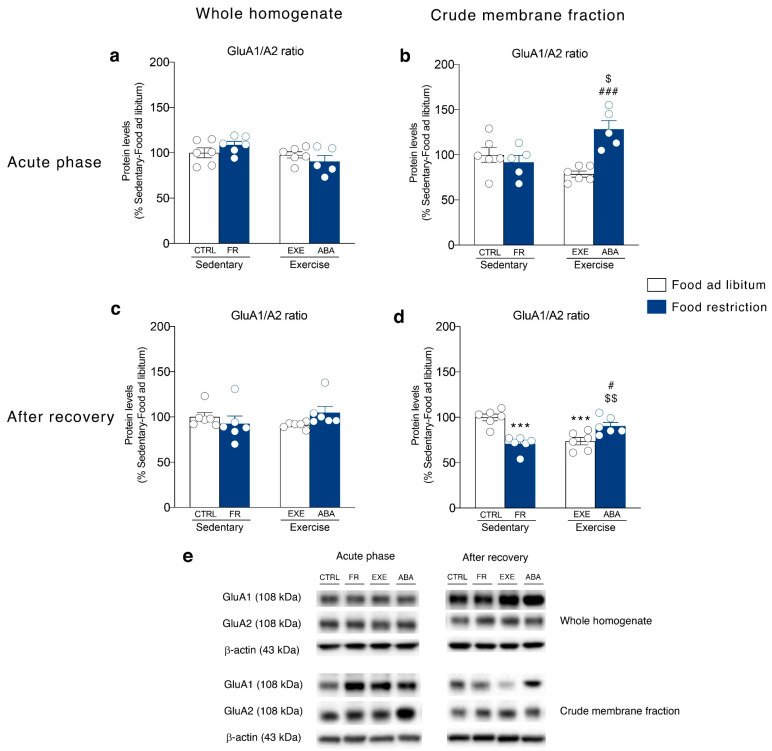
Effect of the ABA induction on the AMPA receptor subunits composition in the whole homogenate (left) and in the crude membrane fraction (right) of the Nucleus Accumbens (NAc) measured in the acute phase of the pathology (postnatal day PND 42) and after a 7-days recovery period (PND 49). Protein levels of GluA1 and GluA2 subunits expressed as GluA1/A2 ratio are shown in the (**a**) homogenate and (**b**) crude membrane fraction at PND 42, and (**c**) in the homogenate and (**d**) crude membrane fraction at PND 49. Results are expressed as percentages of controls and represent the mean ± SEM of five-six rats per group. Panel (**e**) shows representative immunoblots for GluA1 and GluA2. *** *p* < 0.001 vs. Food ad libitum-sedentary; ^$^ *p* < 0.05, ^$$^ *p* < 0.01 vs. Food restriction-sedentary, ^#^ *p* < 0.05, ^###^ *p* < 0.001 vs. Food ad libitum-exercise (two-way ANOVA followed by Tukey’s multiple comparisons test). CTRL = control; FR = food-restricted; EXE = exercise; AMPA: (α-amino-3-hydroxy-5-methyl-4-isoxazolepropionic acid); ABA = activity-based anorexia.

First, since experience-dependent alterations in the accumbal glutamate synapse involve changes in the signaling mediated by AMPA-type receptors [24], we first investigated the GluA1/A2 ratio, which is widely accepted as an indirect index of maladaptive plasticity and incubation of drug craving [26]. In the NAc crude membrane fraction, GluA1/A2 ratio was markedly increased only in ABA rats in the acute phase of the pathology (Figure 3b). After a 7-days period of body weight recovery, the combination of food restriction and exercise increased GluA1/A2 ratio (Figure 3d), whereas when these two conditions were administered separately they reduced such ratio. No changes in AMPA receptor subunit composition were observed in the homogenate at both time points (Figure 3a,c).

**Figure 4:**
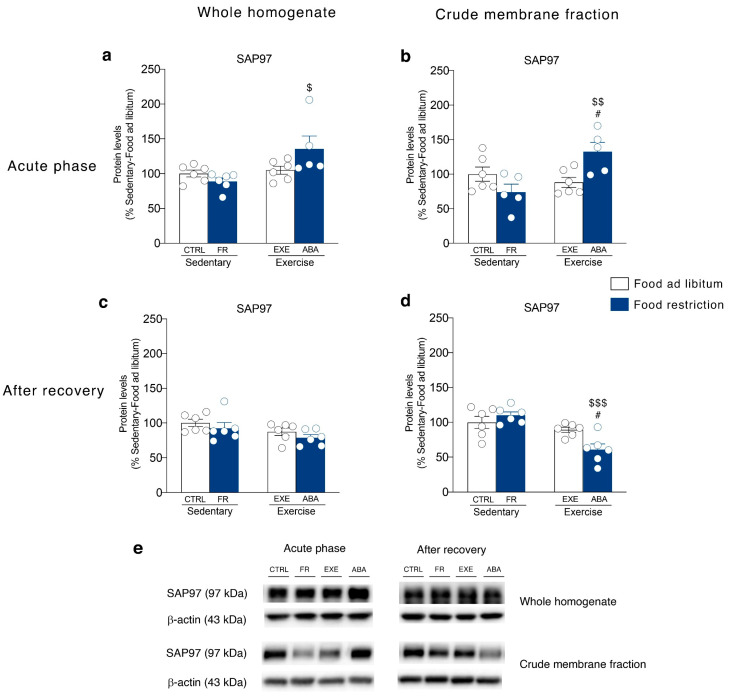
Effect of the ABA induction on SAP97 scaffolding protein expression in the whole homogenate (left) and in the crude membrane fraction (right) of the NAc measured in the acute phase of the pathology (PND 42) and after a 7-days recovery period (PND 49). Protein levels of SAP97 are shown in the (**a**) homogenate and (**b**) crude membrane fraction at PND 42, and in the (**c**) homogenate and (**d**) crude membrane fraction at PND 49. Results are expressed as percentages of controls and represent the mean ± SEM of five-six rats per group. Panel (**e**) shows representative immunoblots for SAP97. ^$^ *p* < 0.05, ^$$^ *p* < 0.01, ^$$$^ *p* < 0.001 vs. Food restriction-sedentary, ^#^ *p* < 0.05 vs. Food ad libitum-exercise (two-way ANOVA followed by Tukey’s multiple comparisons test). CTRL = control; FR = food-restricted; EXE = exercise; ABA = activity-based anorexia.

**Figure 5:**
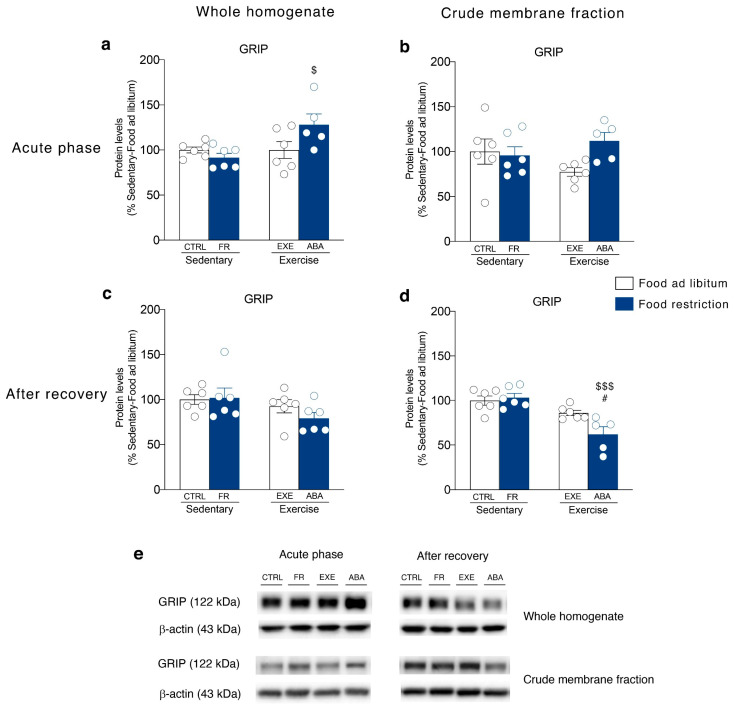
Effect of the ABA induction on GRIP scaffolding protein expression in the whole homogenate (left) and in the crude membrane fraction (right) of the NAc in the acute phase of the pathology (PND 42) and after a 7-days recovery period (PND 49). Protein levels of GRIP are shown in the (**a**) homogenate and (**b**) crude membrane fraction at PND 42, and in the (**c**) homogenate and (**d**) crude membrane fraction at PND 49. Results are expressed as percentages of controls and represent the mean ± SEM of five-six rats per group. Panel (**e**) shows representative immunoblots for GRIP. ^$^ *p* < 0.05, ^$$$^ *p* < 0.001 vs. Food restriction-sedentary, ^#^ *p* < 0.05 vs. Food ad libitum-exercise (two-way ANOVA followed by Tukey’s multiple comparisons test). CTRL = control; FR = food-restricted; EXE = exercise; ABA = activity-based anorexia.

Then, we investigated SAP97 and GRIP levels, the main scaffolding proteins of GluA1 and GluA2 AMPA subunits, i.e., those proteins that anchor the receptors to the membrane and that are also critical for synaptic localization of newly synthesized receptor towards dendritic spines [37]. In the acute phase of the pathology, ABA induction increased SAP97 levels in both homogenate (Figure 4a) and membrane fraction (Figure 4b) whereas GRIP levels in the homogenate (Figure 5a) but not in the membrane fraction (Figure 5b). On the contrary, bodyweight recovery reduced SAP97 and GRIP levels only in the membrane fraction of ABA rats (Figure 4d and Figure 5d), whereas no changes were observed in the homogenate (SAP97: Figure 4c; GRIP: Figure 5c).

**Figure 6:**
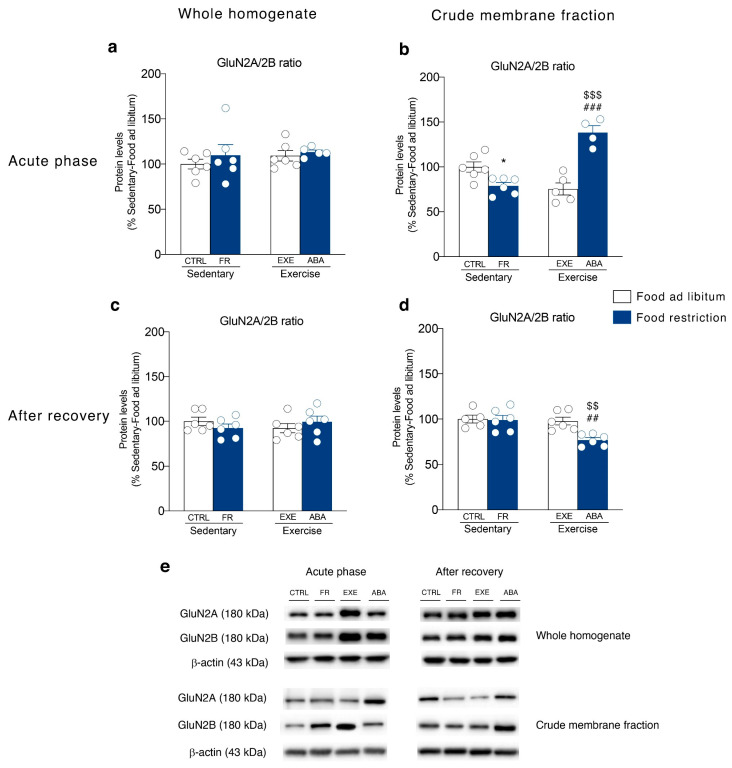
Effect of the ABA induction on the NMDA receptor subunits composition in the whole homogenate (left) and in the crude membrane fraction (right) of the NAc in the acute phase of the pathology (PND 42) and after a 7-days recovery period (PND 49). Protein levels of GluN2A and GluN2B receptors expressed as GluN2A/2B ratio are shown in the (**a**) homogenate and (**b**) crude membrane fraction at PND 42, and in the (**c**) homogenate and (**d**) crude membrane fraction at PND 49. Results are expressed as percentages of controls and represent the mean ± SEM of five-six rats per group. Panel (**e**) shows representative immunoblots for GluN2A and GluN2B. * *p* < 0.05 vs. Food ad libitum-sedentary, ^$$^ *p* < 0.01, ^$$$^ *p* < 0.001 vs. Food restriction-sedentary, ^##^ *p* < 0.01, ^$$$^ *p* < 0.001 vs. Food restriction-sedentary, ^###^ *p* < 0.001 vs. Food ad libitum-exercise (two-way ANOVA followed by Tukey’s multiple comparisons test). CTRL = control; FR = food-restricted; EXE = exercise; ABA = activity-based anorexia.

**Figure 7:**
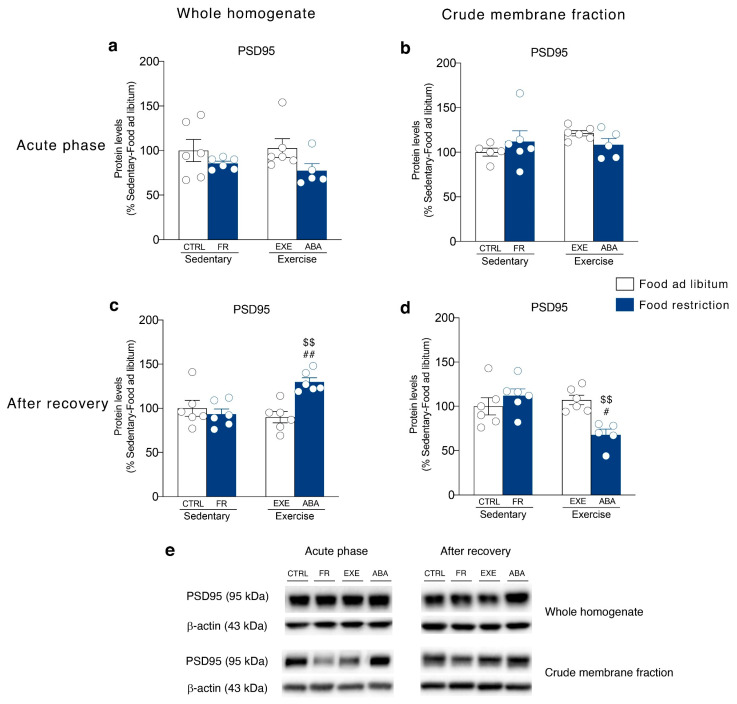
Effect of the ABA induction on PSD95 scaffolding protein expression in the whole homogenate (left) and in the crude membrane fraction (right) of the NAc in the acute phase of the pathology (PND 42) and after a 7-days recovery period (PND 49). Protein levels of PSD95 are shown in the (**a**) homogenate and (**b**) crude membrane fraction at the PND 42, and in (**c**) the homogenate and (**d**) crude membrane fraction at PND 49. Results are expressed as percentages of controls and represent the mean ± SEM of five-six rats per group. Panel (**e**) shows representative immunoblots for PSD95. ^$$^ *p* < 0.01 vs. Food restriction-sedentary, ^#^ *p* < 0.05, ^##^ *p* < 0.01 vs. Food ad libitum-exercise (two-way ANOVA followed by Tukey’s multiple comparisons test). CTRL = control; FR = food-restricted; EXE = exercise; ABA = activity-based anorexia.

**Figure 8:**
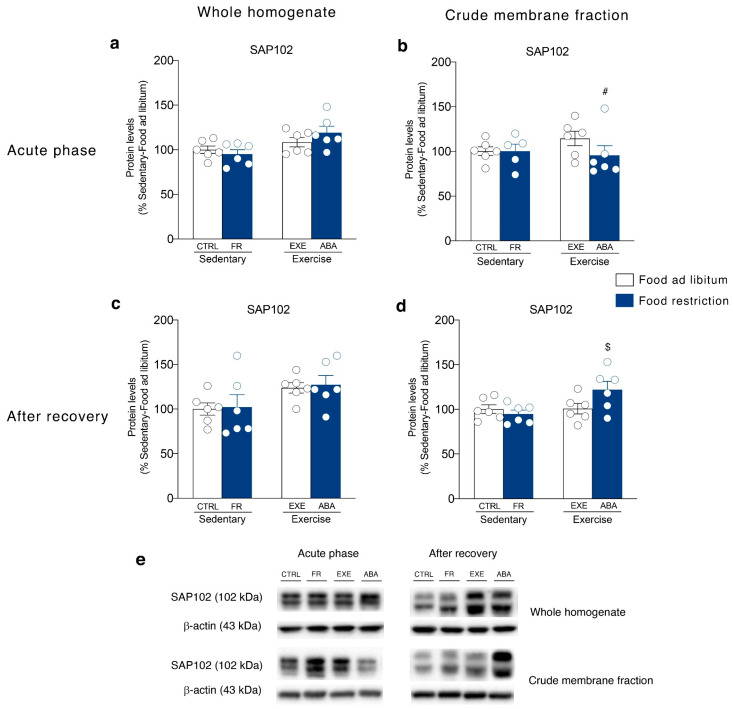
Effect of the ABA induction on SAP102 scaffolding protein expression in the whole homogenate (left) and in the crude membrane fraction (right) of the NAc in the acute phase of the pathology (PND 42) and after a 7-days recovery period (PND 49). Protein levels of SAP102 are shown in the (**a**) homogenate and (**b**) crude membrane fraction at PND 42, and in the (**c**) homogenate and (**d**) crude membrane fraction at PND 49. Results are expressed as percentages of controls and represent the mean ± SEM of five-six rats per group. Panel (**e**) shows representative immunoblots for SAP102. ^$^ *p* < 0.05 vs. Food restriction-sedentary, ^#^ *p* < 0.05 vs. Food ad libitum-exercise (two-way ANOVA followed by Tukey’s multiple comparisons test). CTRL = control; FR = food-restricted; EXE = exercise; ABA = activity-based anorexia.

It has been shown that NMDA subunit composition is plastic, during development, in response to neuronal activity and sensory experience [38]. Moreover, a switch between GluN2A- and GluN2B-containing NMDA receptors correlated with hunger-evoked exercise [39] and may alter the progression of ABA [40]. Food restriction differently altered GluN2A/2B balance in sedentary or hyperactive rats in the acute phase of the pathology. In particular, GluN2A/2B ratio is reduced in the membrane fraction of FR rats whereas increased in ABA rats (Figure 6b). On the contrary, bodyweight recovery reduced the GluN2A/2B ratio only in the ABA recovered rats (Figure 6d). No changes in NMDA receptor subunit composition were observed in the homogenate at both time points (Figure 6a,c). The analysis of the expression levels of PSD95, a scaffolding protein of NMDA receptor and an index of postsynaptic density integrity [31,41], revealed only an increase in the homogenate (Figure 7c) and a reduction in the membrane fraction of ABA recovered rats (Figure 7d). In the acute phase of the pathology, no changes were observed in both homogenate and membrane fraction (Figure 7a,b, respectively). SAP102, the protein that anchor the NMDA receptor in the membrane [42], is reduced in the acute phase (Figure 8b), while increased after recovery (Figure 8d), in the crude membrane fraction of ABA rats. No changes were observed in the homogenate at both time points (Figure 8a,c).

### 3.3. Food Restriction-Evoked Hyperactivity Correlated with GluA1/A2 Ratio

**Figure 9:**
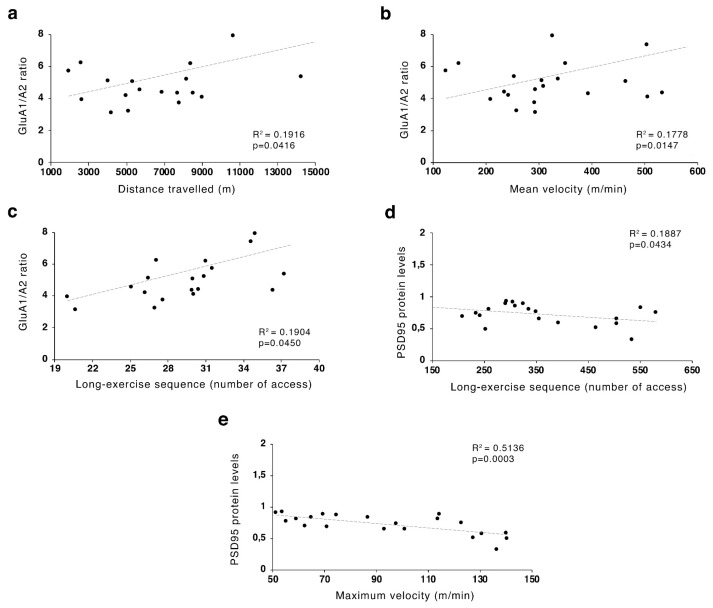
Pearson’s product–moment correlation (*r*) analyses between GluA1/A2 ratio and (**a**) distance travelled, (**b**) mean speed and (**c**) long-exercise sequences and between PSD95 and (**d**) long-exercise sequences and (**e**) maximum speed of ABA and EXE rats. EXE = exercise; ABA = activity-based anorexia.

Pearson correlation analyses were run to determine the relationship between FR-induced hyperactivity and the herein investigated molecular markers in the NAc of ABA rats at both time points. Pearson’s product–moment correlation was generated to correlate total distance travelled, mean and maximum speed on the wheel, long- and brief-exercise sequences with molecular results obtained from GluA1/A2, GluN2A/2B ratio and their scaffolding proteins analysis in the membrane fraction. As shown in Figure 9, GluA1/A2 ratio positively correlated with total distance travelled (Figure 9a), mean speed (Figure 9b) and long-exercise sequences (Figure 9c) whereas PSD95 levels negatively correlated with maximum speed (Figure 9e) and long-exercise sequences (Figure 9d).

## 4. Discussion

We here demonstrate that food restriction (and subsequent malnutrition) leads to hyperactive and compulsive running behavior in female adolescent rats. This rapid escalation of maladaptive behaviors correlates with glutamate receptor subunit redistribution and, ultimately, to an altered molecular composition of the glutamatergic synapse in the NAc. Moreover, the recovery of body weight occurs despite the switch in the AMPA and NMDA subunit composition is still present, indicating that such dysregulation of the glutamate system lasts longer. These ABA-induced changes may represent an aberrant form of neuroplasticity that drives long-term adaptations in the reward circuit, revealing a neurobiological underpinning of altered processing of food reward and a potential trigger for the motivational mechanisms underlying AN.

Our findings revealed that the food restriction is permissive toward the switch from hedonic training into intense and compulsive activity: in fact, we found that mean and maximum speed on the wheel of ABA rats are increased, indicative of an enhanced motivation to run. Moreover, not only ABA rats increased exponentially the daily distance travelled on the wheel, but they voluntarily entered the wheel to perform long-term exercise sequences, demonstrating that low caloric intake drives animals toward a high level of physical activity [43]. Despite the fact that the ABA model does not fully address the complex aspects of AN in humans, our data contribute to demonstrate that this experimental model is a unique tool to investigate the neurobiological underpinning of AN-induced hyperactive behavior. Since intense and compulsive physical activity observed in AN patients interferes with their full recovery and it is also an index of a higher risk of relapse after recovery [10,44], the comprehension of the molecular mechanisms underlying this AN-induced feature is fundamental to better understand the development and maintenance of AN [8,45].

In this scenario, our experiments revealed that the glutamate system is dysregulated in the NAc of ABA rats and the reorganization of the glutamate synapse correlates with ABA-induced hyperactive behavior. In particular, the induction of the ABA phenotype, but not food-restriction or exercise alone, increased the GluA1/A2 ratio in the membrane, an index of GluA2-lacking Ca^2+^-permeable AMPA (CP-AMPA) receptors formation [46]. At the achievement of the anorexic phenotype, this effect was paralleled by increased expression (whole homogenate) and retention in the membrane (crude membrane fraction) of SAP97, the scaffolding protein pivotal for synaptic localization of GluA1 subunits of AMPA receptor [47]. Moreover, despite the translation of GRIP, the scaffolding protein specific for GluA2 AMPA subunit, was enhanced in ABA rats, no changes in its synaptic expression were observed, indicating specific delivery and increased synaptic stability of GluA1-, but not GluA2-containing, CP-AMPA receptors at the membrane. Since increased membrane insertion of CP-AMPA receptors may mediate excitotoxicity [48], redirect the synapse functionality versus immature synapse [49] and promote incubation of drug craving [26], the alterations of the glutamate system herein observed in ABA rats might contribute by influencing synaptic transmission, ultimately driving maladaptive reward-related behaviors typical of AN patients [15,50]. After 7 days of recovery, restoration from the ABA condition leads to a persistent increase in GluA1/A2 ratio, thus confirming the increased formation of CP-AMPA receptors in the NAc as a molecular signature of the combination of starvation and hyperactivity. Notably, the observed increase in the GluA1/A2 ratio was not accompanied by increased expression of the anchoring proteins SAP97 and GRIP, thereby suggesting a reduction in the AMPA receptor stability at the glutamatergic synapse in the NAc. Akin to previous data showing that food-restriction alters NAc synaptic transmission via increased surface expression of GluA1, but not GluA2, subunit [27], our data add complexity as well as specificity to the herein shown reorganization of the AMPA receptor subunit demonstrating that also the synaptic machinery that might anchor these receptors in the membrane is persistently modified by ABA induction.

In concomitance with AMPA receptor reorganization, the ABA phenotype increased the membrane levels of the NMDA GluN2A/2B subunit ratio in the acute phase while reducing it after the recovery of body weight. As observed for AMPA receptors, the lack of effects in the whole homogenate implies that the combination of food restriction and hyperactivity has not influenced the translation of the glutamate receptors but, rather, their subunit reorganization and synaptic retention. During neonatal development, the GluN2A subunit gradually increases while the GluN2B expression progressively decreases: this developmental switch drives changes in synaptic transmission leading to mature synapse formation [51]. Experiences, such as addictive drug exposure [48,52] or ABA induction, are likely to reverse the switch toward GluN2B-containing NMDA receptors, thus re-opening a critical period of vulnerability and re-programming the accumbal excitatory synapse toward immature or silent synapse. Besides, since NMDA receptor-dependent plasticity is required for learning association between environmental cues and reward-related behaviors [53], this phasic plasticity of NMDA receptor subunits observed in the NAc of ABA rats suggests that the consequent potential alteration in neurotransmission might be a signature of the ABA-induced vulnerability. Interestingly, in the acute phase of the pathology, despite the increase in GluN2A/2B ratio in the membrane fraction both anchoring proteins specific for NMDA receptor, SAP102 and PSD95, were reduced, thus suggesting NMDA receptor instability and altered NMDA-dependent neurotransmission. In addition, the reduced expression of PSD-95, a postsynaptic protein required for normal synaptic transmission of both AMPA and NMDA receptors [41], in the membrane fraction, but not in the whole homogenate, further supports the hypothesis of impaired synaptic plasticity in the NAc of ABA rats. The reduced GluN2A/2B ratio observed despite body weight recovery might be considered as an index of vulnerability trait of ABA rats since it has been demonstrated that GluN2A knockout mice display an increased baseline physical activity and, when subjected to the ABA model, these mice lost significantly more weight than their wild-type littermates [40]. Moreover, our data are in line with the previously observed correlation between ABA severity and increased GluN2B-containing NMDA receptor observed in the postsynaptic terminal of the hippocampus [39].

Taken together, we demonstrate that low caloric intake is sufficient to compulsively increase physical activity in female adolescent rats, a core feature of AN pathology. From a neurobiological point of view, our findings, showing a switch toward GluA2-lacking and GluN2B-containing receptors, are reminiscent of the neuroadaptive changes of the glutamate synapse that are usually seen after chronic exposure to drugs of abuse [25,48]. In fact, as observed for drugs of abuse, we hypothesized that ABA induction via reorganization of the glutamatergic synapse might re-open a critical period of vulnerability, during which changes in functional properties of glutamate receptors might influence synaptic transmission, remodel reward-related neurocircuitry and alter food- and exercise-related behavior. We are aware that our work is limited by the lack of electrophysiological measures to substantiate our hypothesis; however, these data are the first to hypothesize that changes in glutamate determinants in the NAc may contribute to the reward mechanisms set in motion by the ABA phenotype.

Since no effective treatments for AN patients are available, our novel data in a rodent model mimicking the human condition might pave the way toward new pharmacological intervention. Our data, in line with the hypothesis that the imbalance between cognitive and reward circuitries likely interferes with motivation for treatment and ability to learn from experience [20] suggest that pharmacotherapies designed to manipulate the glutamate system might enable ill individuals to stop the vicious cycle of the disease.

## Supplementary Materials

The following are available online at https://www.mdpi.com/2072-6643/12/12/3661/s1, Table S1: Repeated measures two-way ANOVA analysis of data presented in Figure 1 and Figure 2, Table S2: Detailed statistical values of data presented in Figure 1. Mean differences and adjusted *p* values relative to Bonferroni’s multiple comparisons test are presented for the analysis of body weight (Figure 1b) and food intake (Figure 1c), Table S3: Detailed statistical values of data presented in Figure 2. Mean differences and adjusted *p* values relative to Bonferroni’s multiple comparisons test are presented for the analysis of distance travelled (Figure 2a), mean speed (Figure 2b), maximum speed (Figure 2c) and long-exercise sequence (Figure 2e), Table S4: Two-way ANOVA analysis of protein expression data measured in the whole homogenate of the NAc in the acute phase of the pathology (right) and after a 7-days recovery period (left) presented in Figure 3, Figure 4, Figure 5, Figure 6, Figure 7 and Figure 8, Table S5: Two-way ANOVA analysis of protein expression data measured in the crude synaptosomal fraction of the NAc in the acute phase of the pathology (right) and after a 7-days recovery period (left) presented in Figure 3, Figure 4, Figure 5, Figure 6, Figure 7 and Figure 8, Figure S1 Uncropped immunoblot related to the expression levels of GluN2A (180 kDa), GluN2B (180 kDa), GRIP (122 kDa), GluA1 (108 kDa), GluA2 (108 kDa), SAP102 (102 kDa), SAP97 (97 kDa), PSD95 (95 kDa), β-actin (43 kDa) measured in the NAc in the whole homogenate of CTRL, FR, EXE and ABA rats in the acute phase of the pathology and after a 7-days recovery period, presented in Figure 3, Figure 4, Figure 5, Figure 6, Figure 7 and Figure 8, Figure S2 Uncropped immunoblot related to the expression levels of GluN2A (180 kDa), GluN2B (180 kDa), GRIP (122 kDa), GluA1 (108 kDa), GluA2 (108 kDa), SAP102 (102 kDa), SAP97 (97 kDa), PSD95 (95 kDa), β-actin (43 kDa) measured in the NAc in the crude membrane fraction of CTRL, FR, EXE and ABA rats in the acute phase of the pathology and after a 7-days recovery period, presented in Figure 3, Figure 4, Figure 5, Figure 6, Figure 7 and Figure 8.
