# Supplementary material for: Activity-Based Anorexia Dynamically Dysregulates the Glutamatergic Synapse in the Nucleus Accumbens of Female Adolescent Rats
Source: Nutrients. 2020 Nov 28;12(12):3661. doi: 10.3390/nu12123661 (PMC7760003; doi:10.3390/nu12123661)

Supplementary Table S1. Repeated measures two-way ANOVA analysis of data presented in Figures 1 and 2

**Two-way ANOVA**	**Body weight (%)**		**Food intake (g)**		**Distance travelled (m)**	
	F (DFn, DFd)	*P value*	*F (DFn, DFd)*	*P value*	*F (DFn, DFd)*	*P value*
Time x treatment	F (54, 360) = 10,54	P<0,0001	F (39, 260) = 32,63	P<0,0001	F (6, 174) = 6,852	P<0,0001
Time	F (18, 360) = 475,5	P<0,0001	F (5,399, 108,0) = 130,2	P<0,0001	F (6, 174) = 41,91	P<0,0001
Food restriction	F (3, 20) = 9,131	P=0,0005	F (3, 20) = 5,316	P=0,0074	F (1, 29) = 20,13	P=0,0001
Exercise	F (20, 360) = 18,35	P<0,0001	F (20, 260) = 5,568	P<0,0001	F (29, 174) = 2,615	P<0,0001

	**Mean speed (m/min)**		**Max speed (m/min)**		**Long-exercise sequence**	
	F (DFn, DFd)	*P value*	*F (DFn, DFd)*	*P value*	*F (DFn, DFd)*	*P value*
Time x treatment	F (6, 126) = 3,113	P=0,0071	F (6, 126) = 10,93	P<0,0001	F (6, 126) = 4,681	P=0,0002
Time	F (6, 126) = 39,90	P<0,0001	F (6, 126) = 21,27	P<0,0001	F (6, 126) = 12,05	P<0,0001
Food restriction	F (1, 21) = 4,334	P=0,0498	F (1, 21) = 4,948	P=0,0372	F (1, 21) = 4,113	P=0,0554
Exercise	F (21, 126) = 5,767	P<0,0001	F (21, 126) = 10,90	P<0,0001	F (21, 126) = 2,932	P=0,0001

Supplementary Table S2. Detailed statistical values of data presented in Figure 1. Mean differences and adjusted *p* values relative to Bonferroni's multiple comparisons test are presented for the analysis of body weight (Figure 1b) and food intake (Figure 1c)

Body weight	Bonferroni's multiple comparisons test	
	Mean difference (g)	Adjusted p value
**pnd39**		
Control vs. Food restriction	18,92	0,0066
Control vs. Activity-based anorexia	22,67	0,0002
Exercise vs. Activity-based anorexia	16,60	0,0461
**pnd40**		
Control vs. Food restriction	30,25	<0,0001
Control vs. Activity-based anorexia	35,29	<0,0001
Food restriction vs. Exercise	-23,37	<0,0001
Exercise vs. Activity-based anorexia	28,42	<0,0001
**pnd41**		
Control vs. Food restriction	36,52	<0,0001
Control vs. Activity-based anorexia	44,28	<0,0001
Food restriction vs. Exercise	-30,54	<0,0001
Exercise vs. Activity-based anorexia	38,30	<0,0001
**pnd42**		
Control vs. Food restriction	37,03	<0,0001
Control vs. Activity-based anorexia	54,81	<0,0001
Food restriction vs. Exercise	-31,43	<0,0001
Food restriction vs. Activity-based anorexia	17,79	0,0174
Exercise vs. Activity-based anorexia	49,22	<0,0001
**pnd43**		
Control vs. Food restriction	17,62	0,0199
Control vs. Exercise	1,663	>0,9999
Control vs. Activity-based anorexia	22,90	0,0001
Food restriction vs. Activity-based anorexia	5,280	>0,9999
Exercise vs. Activity-based anorexia	21,24	0,0008
**pnd44**		
Control vs. Exercise	2,224	>0,9999
Control vs. Activity-based anorexia	19,86	0,0028
Food restriction vs. Exercise	-11,96	>0,9999
Food restriction vs. Activity-based anorexia	5,681	>0,9999
Exercise vs. Activity-based anorexia	17,64	0,0197
**pnd45**		
Control vs. Exercise	0,7040	>0,9999
Control vs. Activity-based anorexia	16,76	0,0405

Food intake	Bonferroni's multiple comparisons test	
	Mean difference (g)	Adjusted p value
**pnd36**		
Control vs. Exercise	3,583	0,0358
**pnd39**		
Control vs. Food restriction	15,06	<0,0001
Control vs. Activity-based anorexia	14,88	<0,0001
Food restriction vs. Exercise	-15,14	<0,0001
Exercise vs. Activity-based anorexia	14,96	<0,0001
**pnd40**		
Control vs. Food restriction	13,36	<0,0001
Control vs. Activity-based anorexia	12,33	<0,0001
Food restriction vs. Exercise	-13,15	<0,0001
Exercise vs. Activity-based anorexia	12,13	<0,0001
**pnd41**		
Control vs. Food restriction	11,52	<0,0001
Control vs. Activity-based anorexia	10,40	<0,0001
Food restriction vs. Exercise	-13,83	<0,0001
Exercise vs. Activity-based anorexia	12,71	<0,0001
**pnd42**		
Control vs. Food restriction	10,77	<0,0001
Control vs. Activity-based anorexia	10,19	0,0003
Food restriction vs. Exercise	-12,63	<0,0001
Exercise vs. Activity-based anorexia	12,04	<0,0001
**pnd43**		
Control vs. Food restriction	-6,167	0,0095
**pnd45**		
Control vs. Food restriction	-4,667	0,0083
**pnd46**		
Control vs. Activity-based anorexia	-6,083	0,0222
**pnd47**		
Control vs. Activity-based anorexia	-7,917	0,0073
Exercise vs. Activity-based anorexia	-6,333	0,0038
**pnd48**		
Control vs. Activity-based anorexia	-4,250	0,0271

Supplementary Table S3. Detailed statistical values of data presented in Figure 2. Mean differences and adjusted *p* values relative to Bonferroni's multiple comparisons test are presented for the analysis of distance travelled (Figure 2a), mean speed (Figure 2b), maximum speed (Figure 2c) and long-exercise sequence (Figure 2e)

**Distance travelled**	Bonferroni's multiple comparisons test	
	Mean difference (m)	Adjusted p value
**Exercise - Activity-based anorexia**		
**pnd40**	-3877	<0,0001
**pnd41**	-4510	<0,0001
**pnd42**	-4351	<0,0001

**Long-exercise sequences**	Bonferroni's multiple comparisons test
	Adjusted p value
**Exercise - Activity-based anorexia**	
**pnd41**	0,0241
**pnd42**	0,0001

**Mean velocity**	Bonferroni's multiple comparisons test
	Adjusted p value
**Exercise - Activity-based anorexia**	
**pnd41**	0,0031
**pnd42**	0,0201

**Max velocity**	Bonferroni's multiple comparisons test
	Adjusted p value
**Exercise - Activity-based anorexia**	
**pnd41**	0,0333
**pnd42**	<0,0001

Supplementary Table S4. Two-way ANOVA analysis of protein expression data measured in the whole homogenate of the NAc in the acute phase of the pathology (right) and after a 7-days recovery period (left) presented in Figures 3–8

	**Two-way ANOVA**	**Whole homogenate – Acute phase**	
		**F (DFn, DFd)**	**P value**
**NR2A**	Interaction	F (1, 19) = 1,431	P=0,2464
	Exercise	F (1, 19) = 12,25	P=0,0024
	Food-restriction	F (1, 19) = 0,2040	P=0,6567
**NR2B**	Interaction	F (1, 19) = 1,377	P=0,2552
	Exercise	F (1, 19) = 8,237	P=0,0098
	Food-restriction	F (1, 19) = 1,014	P=0,3266
**GRIP**	Interaction	F (1, 19) = 5,600	P=0,0287
	Exercise	F (1, 19) = 5,535	P=0,0296
	Food-restriction	F (1, 19) = 1,602	P=0,2209
**GluA1**	Interaction	F (1, 19) = 0,6317	P=0,4365
	Exercise	F (1, 19) = 0,9214	P=0,3492
	Food-restriction	F (1, 19) = 0,006563	P=0,9363
**GluA2**	Interaction	F (1, 19) = 2,761	P=0,1130
	Exercise	F (1, 19) = 5,560	P=0,0292
	Food-restriction	F (1, 19) = 0,002572	P=0,9601
**SAP102**	Interaction	F (1, 19) = 1,004	P=0,3289
	Exercise	F (1, 19) = 7,228	P=0,0145
	Food-restriction	F (1, 19) = 0,0005493	P=0,9815
**SAP97**	Interaction	F (1, 19) = 4,999	P=0,0376
	Exercise	F (1, 19) = 7,629	P=0,0124
	Food-restriction	F (1, 19) = 1,096	P=0,3083
**PSD95**	Interaction	F (1, 19) = 0,3133	P=0,5822
	Exercise	F (1, 19) = 0,08531	P=0,7734
	Food-restriction	F (1, 19) = 4,535	P=0,0465

	**Two-way ANOVA**	**Whole homogenate – Recovery phase**	
		**F (DFn, DFd)**	**P value**
**NR2A**	Interaction	F (1, 20) = 22,44	P=0,0001
	Exercise	F (1, 20) = 11,66	P=0,0027
	Food-restriction	F (1, 20) = 9,209	P=0,0065
**NR2B**	Interaction	F (1, 20) = 28,01	P<0,0001
	Exercise	F (1, 20) = 30,03	P<0,0001
	Food-restriction	F (1, 20) = 27,06	P<0,0001
**GRIP**	Interaction	F (1, 20) = 0,9104	P=0,3514
	Exercise	F (1, 20) = 3,692	P=0,0690
	Food-restriction	F (1, 20) = 0,5258	P=0,4768
**GluA1**	Interaction	F (1, 20) = 20,19	P=0,0002
	Exercise	F (1, 20) = 0,3298	P=0,5722
	Food-restriction	F (1, 20) = 3,079	P=0,0946
**GluA2**	Interaction	F (1, 20) = 1,697	P=0,2074
	Exercise	F (1, 20) = 0,6153	P=0,4420
	Food-restriction	F (1, 20) = 7,024	P=0,0154
**SAP102**	Interaction	F (1, 20) = 0,003614	P=0,9527
	Exercise	F (1, 20) = 6,177	P=0,0219
	Food-restriction	F (1, 20) = 0,07693	P=0,7843
**SAP97**	Interaction	F (1, 20) = 0,008498	P=0,9275
	Exercise	F (1, 20) = 4,665	P=0,0431
	Food-restriction	F (1, 20) = 1,885	P=0,1849
**PSD95**	Interaction	F (1, 20) = 12,40	P=0,0021
	Exercise	F (1, 20) = 3,846	P=0,0640
	Food-restriction	F (1, 20) = 6,445	P=0,0195

Supplementary Table S5. Two-way ANOVA analysis of protein expression data measured in the crude synaptosomal fraction of the NAc in the acute phase of the pathology (right) and after a 7-days recovery period (left) presented in Figures 3–8

	Two-way ANOVA	Crude membrane fraction – Acute phase	
		F (DFn, DFd)	P value
**NR2A**	Interaction	F (1, 17) = 17,12	P=0,0007
	Exercise	F (1, 17) = 26,07	P<0,0001
	Food-restriction	F (1, 17) = 32,25	P<0,0001
**NR2B**	Interaction	F (1, 18) = 15,78	P=0,0009
	Exercise	F (1, 18) = 2,986	P=0,1011
	Food-restriction	F (1, 18) = 0,5549	P=0,4659
**GRIP**	Interaction	F (1, 19) = 3,625	P=0,0722
	Exercise	F (1, 19) = 0,1049	P=0,7495
	Food-restriction	F (1, 19) = 2,240	P=0,1509
**GluA1**	Interaction	F (1, 18) = 2,970	P=0,1020
	Exercise	F (1, 18) = 3,913	P=0,0634
	Food-restriction	F (1, 18) = 6,350	P=0,0214
**GluA2**	Interaction	F (1, 19) = 35,47	P<0,0001
	Exercise	F (1, 19) = 0,9781	P=0,3351
	Food-restriction	F (1, 19) = 0,6139	P=0,4430
**SAP102**	Interaction	F (1, 18) = 5,283	P=0,0337
	Exercise	F (1, 18) = 0,003415	P=0,9540
	Food-restriction	F (1, 18) = 5,000	P=0,0382
**SAP97**	Interaction	F (1, 18) = 10,99	P=0,0039
	Exercise	F (1, 18) = 4,814	P=0,0416
	Food-restriction	F (1, 18) = 0,7498	P=0,3979
**PSD95**	Interaction	F (1, 18) = 2,580	P=0,1257
	Exercise	F (1, 18) = 1,272	P=0,2741
	Food-restriction	F (1, 18) = 0,0009369	P=0,9759

	Two-way ANOVA	Crude membrane fraction – Recovery phase	
		F (DFn, DFd)	P value
**NR2A**	Interaction	F (1, 20) = 22,44	P=0,0001
	Exercise	F (1, 20) = 11,66	P=0,0027
	Food-restriction	F (1, 20) = 9,209	P=0,0065
**NR2B**	Interaction	F (1, 20) = 28,01	P<0,0001
	Exercise	F (1, 20) = 30,03	P<0,0001
	Food-restriction	F (1, 20) = 27,06	P<0,0001
**GRIP**	Interaction	F (1, 19) = 6,378	P=0,0206
	Exercise	F (1, 19) = 26,20	P<0,0001
	Food-restriction	F (1, 19) = 3,669	P=0,0706
**GluA1**	Interaction	F (1, 20) = 20,19	P=0,0002
	Exercise	F (1, 20) = 0,3298	P=0,5722
	Food-restriction	F (1, 20) = 3,079	P=0,0946
**GluA2**	Interaction	F (1, 20) = 1,697	P=0,2074
	Exercise	F (1, 20) = 0,6153	P=0,4420
	Food-restriction	F (1, 20) = 7,024	P=0,0154
**SAP102**	Interaction	F (1, 20) = 4,475	P=0,0471
	Exercise	F (1, 20) = 4,731	P=0,0418
	Food-restriction	F (1, 20) = 1,651	P=0,2135
**SAP97**	Interaction	F (1, 20) = 8,141	P=0,0098
	Exercise	F (1, 20) = 20,05	P=0,0002
	Food-restriction	F (1, 20) = 1,890	P=0,1844
**PSD95**	Interaction	F (1, 19) = 11,28	P=0,0033
	Exercise	F (1, 19) = 5,877	P=0,0255
	Food-restriction	F (1, 19) = 3,221	P=0,0886

Supplementary Figure S1. Uncropped immunoblot related to the expression levels of GluN2A (180 kDa), GluN2B (180 kDa), GRIP (122 kDa), GluA1 (108 kDa), GluA2 (108 kDa), SAP102 (102 kDa), SAP97 (97 kDa), PSD95 (95 kDa), β-actin (43 kDa) measured in the NAc in the whole homogenate of CTRL, FR, EXE and ABA rats in the acute phase of the pathology and after a 7-days recovery period, presented in Figures 3–8 .
CTRL = control; FR = food-restricted; EXE = exercise; ABA = activity-based anorexia.

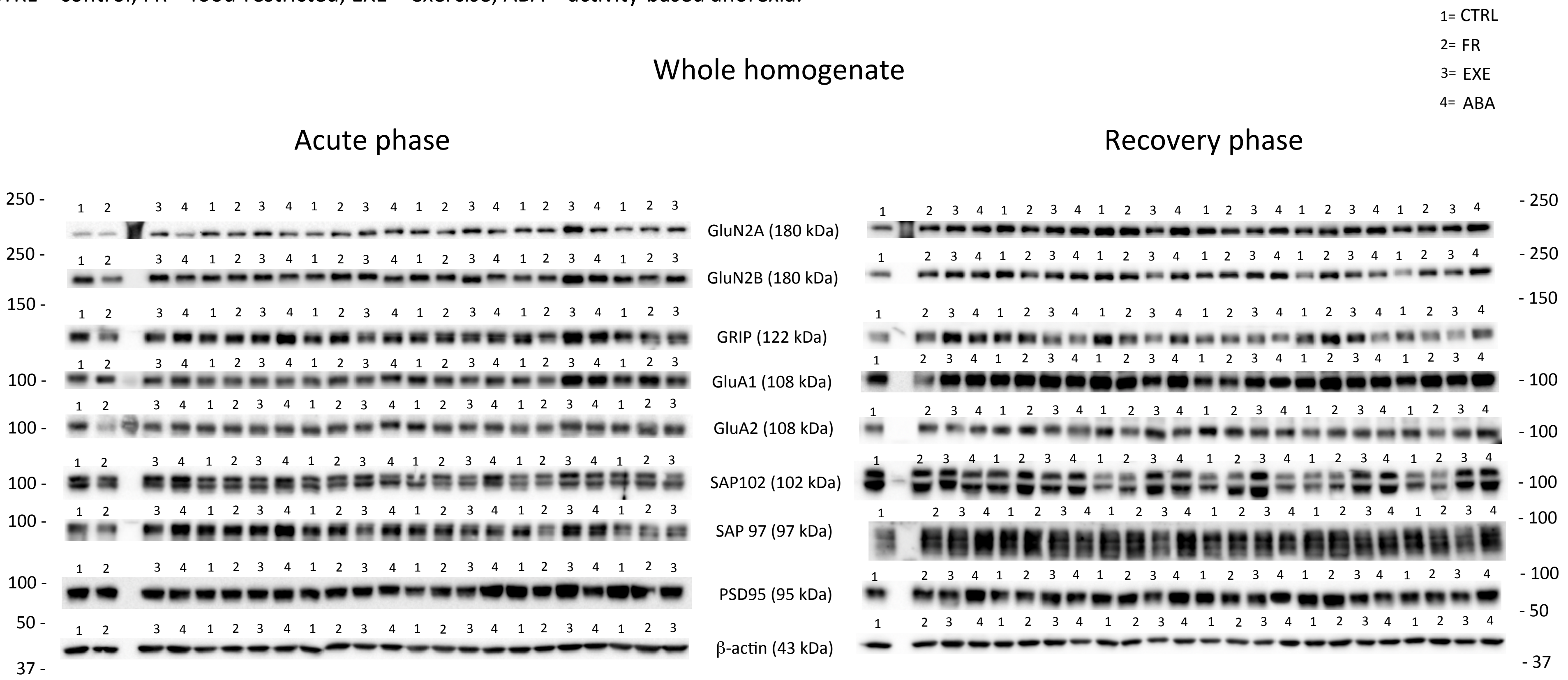

Supplementary Figure S2. Uncropped immunoblot related to the expression levels of GluN2A (180 kDa), GluN2B (180 kDa), GRIP (122 kDa), GluA1 (108 kDa), GluA2 (108 kDa), SAP102 (102 kDa), SAP97 (97 kDa), PSD95 (95 kDa), β-actin (43 kDa) measured in the NAc in the crude membrane fraction of CTRL, FR, EXE and ABA rats in the acute phase of the pathology and after a 7-days recovery period, presented in Figures 3–8.
CTRL = control; FR = food-restricted; EXE = exercise; ABA = activity-based anorexia.

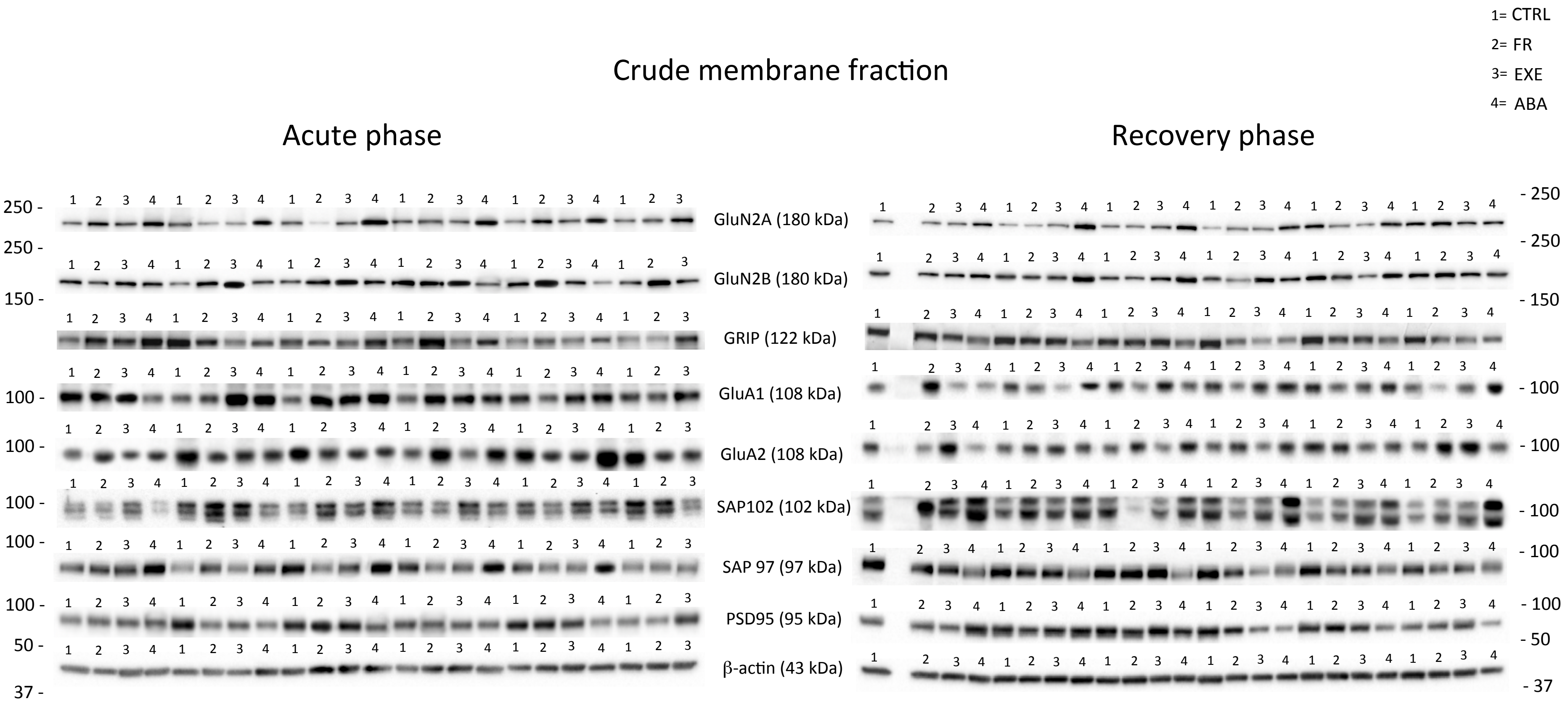

Supplement: Supplementary file 1 [file nutrients-12-03661-s001.pdf]
